# Population Genetic Structure and Biogeographic Distribution of Tropical *Halodule uninervis* in the Bohol Sea and Adjacent Waters in the Philippines

**DOI:** 10.1002/ece3.73727

**Published:** 2026-05-24

**Authors:** Angela Grace E. Singson, Koji Takayama, Yoshihisa Suyama, Naoko Ishikawa, Shoki Murakami, Venus E. Leopardas, Nonillon M. Aspe, Wilfredo H. Uy, Lilibeth P. Coronel, Dan M. Arriesgado, Ruby C. Gonzales

**Affiliations:** ^1^ Mindanao State University at Naawan Naawan Misamis Oriental Philippines; ^2^ Department of Fisheries, College of Agriculture and Forestry Central Philippines State University Ilog Negros Occidental Philippines; ^3^ Makino Herbarium, Department of Biological Science, Graduate School of Science Tokyo Metropolitan University Tokyo Japan; ^4^ Kawatabi Field Science Center, Graduate School of Agricultural Science Tohoku University Osaki Miyagi Japan

**Keywords:** coastal management, geographic connectivity, population genetics, restoration, seagrass, single‐nucleotide polymorphism (SNP)

## Abstract

*Halodule uninervis* plays a critical role in the seagrass ecosystem because its opportunistic traits facilitate expansion and persistence under changing environmental conditions. Yet the population genetic structure and connectivity of this species in the Philippines remain poorly understood. Using genome‐wide single‐nucleotide polymorphisms (6952 SNPs) generated from MIG‐seq data, we assessed clonal reproduction, genetic diversity, population structure, isolation by distance, and recent migration across 10 populations in the Bohol Sea and adjacent waters. Clone detection revealed pronounced spatial variation ranging from predominantly sexual populations with high genotypic richness to populations with strongly clonal populations dominated by relatively few multilocus genotypes. Genetic diversity was generally low to moderate across populations. A geographically separated population from the Visayan Sea exhibited comparatively higher nucleotide diversity and distinct ancestry patterns relative to most Bohol Sea populations. Population structure analyzes showed weak but detectable genetic structuring, with the first two principal components explaining 6.3% and 5.1% of the total variance. ADMIXTURE analyzes showed partially shared ancestry profiles among several populations. Genetic differentiation was low to moderate, with no significant association between geographic distances. These results suggest that geographic distance alone may not fully explain connectivity patterns within the study area. Recent migration analyzes revealed generally high self‐recruitment together with asymmetric migration estimates among several populations, including comparatively higher incoming migration into Mambajao. These overall findings suggest a partially connected population dynamics influenced by clonal reproduction, localized recruitment, and regional dispersal processes. This study provides baseline genetic information that may support future conservation, monitoring, and restoration planning for tropical seagrass ecosystems in the Philippines.

## Graphical abstract

Genome‐wide SNP markers reveal patterns of genetic structure and connectivity among *Halodule uninervis* populations across the Bohol Sea. Migration rates (≥ 0.025 and ≤ 0.040) indicate asymmetric gene flow and heterogeneous connectivity, suggesting a semi‐connected metapopulation shaped by clonal reproduction and regional oceanographic circulation.

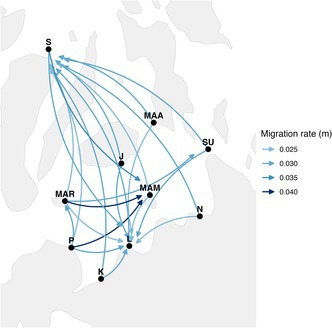

## 1 Introduction

One of the diverse seagrass species belongs to the genus *Halodule*. *Halodule* species have less robust rhizomes and limited tolerance to prolonged low‐light conditions due to relatively low carbohydrate reserves and weak clonal integration compared to other species, which makes them susceptible to physical disturbances, including strong currents and turbulence (Uy 2001; McKenzie et al. 2021). Despite this, *Halodule* species are fast‐growing pioneer species capable of rapid colonization through sexual reproduction and clonal expansion, contributing to habitat recovery and resilience in dynamic coastal environments (Uy 2001; McKenzie et al. 2021).

In the Philippines, population genetic studies have predominantly focused on the dominant seagrass species, including *Cymodecea*, *Enhalus*, *Thalassia*, and with limited inclusion of *Syringodium* spp. (e.g., Nakajima et al. 2014a, 2014b, 2023; Arriesgado, Kurokochi, et al. 2016; Arriesgado, Uy, et al. 2016; Arriesgado et al. 2023a, 2023b; Kurokochi et al. 2016; Malanguis et al. 2024). As a result, the population structure and genetic connectivity of *Halodule uninervis* remain largely unexplored despite their wide distribution across Southeast Asia, particularly in diverse Philippine coastal habitats (Singson et al. 2025). Genetic diversity and population genetic structure are essential factors in understanding the resilience, adaptation and connectivity, particularly in marine systems where dispersal is influenced by both biological traits and oceanographic processes (Pazzaglia et al. 2021; Hernawan et al. 2023; Hosokawa et al. 2025).

The Bohol Sea represents a is a complex and interconnected seascape bounded by the Visayas and Mindanao islands, characterized by dynamic oceanographic processes including the Bohol Jet and localized eddies (Cabrera et al. 2011; Gordon et al. 2011). These features have the potential to facilitate long‐distance dispersal and influence genetic connectivity among marine populations. However, genetic studies have primarily focused on dominant seagrass species, and no studies have explicitly examined the population genetics and connectivity *of* *H. uninervis* within the Bohol Sea.

The extent to which geographic distance versus oceanographic processes shape genetic differentiation in *H. uninervis* populations in this region remains unclear. If dispersal is primarily limited by distance, populations are expected to exhibit isolation by distance (IBD), with increasing genetic differentiation over space. In contrast, if oceanographic processes facilitate connectivity, genetic structure may be weak and not strongly associated with geographic distance, reflecting high levels of gene flow across the seascape.

In addition to these ecological questions, genetic information remains underutilized in seagrass restoration efforts in the Philippines. Current practices often emphasize physicochemical compatibility between donor and recipient sites, with limited consideration of genetic diversity and connectivity (e.g., Creencia et al. 2023). Incorporating genetic data is essential to improve restoration success, as it informs the selection of populations, enhances adaptive potential, and supports long‐term population persistence.

Given these knowledge gaps, this study investigates the population genetic structure and connectivity of *H. uninervis* across the Bohol Sea and adjacent areas. Specifically, it assesses the clonal and genetic diversity, population genetic structure and differentiation, isolation by distance, and migration patterns across the region. This study provides insights into the processes shaping connectivity and offers baseline genetic information to support conservation and restoration strategies in seagrass ecosystems.

## 2 Materials and Methods

### 2.1 Sampling Sites

**FIGURE 1:**
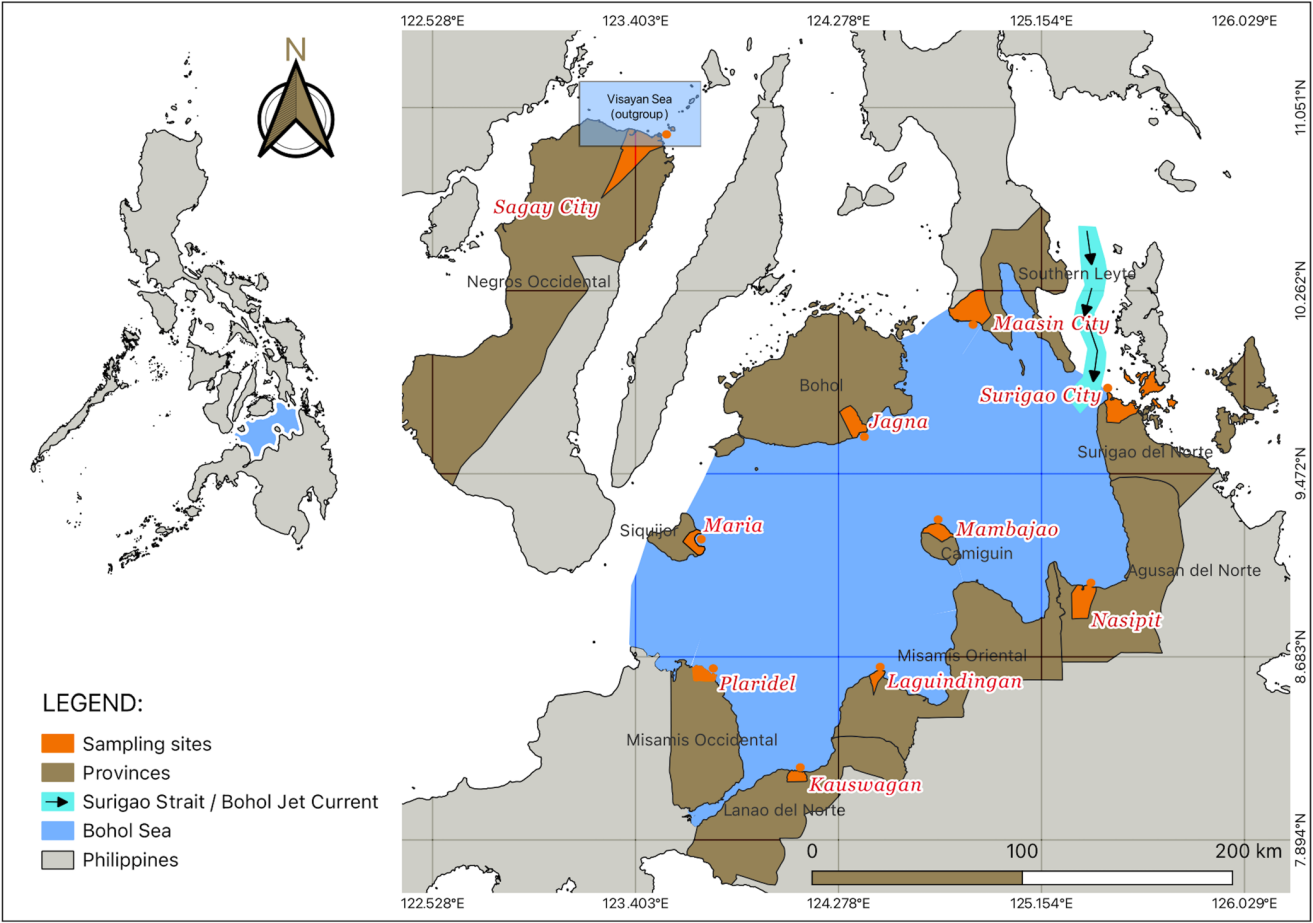
The nine study sites are within the Bohol Sea, and one is in the Visayan Sea. The map was generated using the Philippine base map in QGIS v3.14. The circulation figure of the Bohol Jet current passing through the Surigao Strait is shown for spatial reference, with basin boundaries defined according to the configuration described by Cabrera et al. (2011).

The study was conducted in Bohol and the Visayan Seas, including Surigao City, Nasipit, Mambajao, Laguindingan, Kauswagan, Plaridel, Maasin City, Jagna, and Maria. To provide a comprehensive comparison of inferences about regional genetic structuring, at least one outgroup population was recommended as a reference. Sagay from the Visayan Sea is geographically separated from the Bohol Sea sites and serves as a comparative baseline for evaluating patterns of genetic differentiation and connectivity (Figure 1; Table 1).

**TABLE 1:** The study sites with their corresponding coordinates.

Study sites	Latitude	Longitude
Surigao City, Surigao del Norte	9.818196° N	125.451416° E
Nasipit, Agusan del Norte	8.991126° N	125.346375° E
Mambajao, Camiguin	9.253386° N	124.722617° E
Laguindingan, Misamis Oriental	8.625000° N	124.465300° E
Kauswagan, Lanao del Norte	8.220400° N	124.111944° E
Plaridel, Misamis Occidental	8.605172° N	123.740517° E
Maasin, Southern Leyte	10.145564° N	124.769739° E
Jagna, Bohol	9.643046° N	124.366302° E
Maria, Siquijor	9.178706° N	123.661102° E
Sagay City, Negros Occidental	11.051089° N	123.456253° E

### 2.2 Sampling Collection and DNA Extraction

At each sampling site, 25 to 28 vegetative shoots were randomly collected, maintaining a 10‐m distance between each sample to avoid overestimating clonal diversity (Arriesgado et al. 2015; Nakajima et al. 2017). The collection area for each site ranged from 200 to 300 m in length and 30 to 40 m in width. Samples were put into tea bags and preserved in silica gel within zip‐lock plastic bags at room temperature. Each silica‐gel‐dried leaf tissue sample (< 10 mg) underwent total genomic DNA extraction using a modified cetyltrimethylammonium bromide (CTAB) protocol following Doyle and Doyle (1987). Minor modifications were introduced to improve the DNA quality from seagrass tissue, including adjustments to incubation time and purification steps to remove polysaccharides.

### 2.3 Multiplexed ISSR Genotyping by Sequencing (MIG‐Seq) Protocol and Procedures

A multiplexed inter‐simpler sequence repeat genotyping‐by‐sequencing (MIG‐seq) library was constructed following the original method of Suyama and Matsuki (2015), with subsequent protocol refinements described in Suyama et al. (2022). The procedure consists of two PCR amplification steps.

The first PCR used a Multiplex PCR Assay Kit Version 2 (Takara RR062) with MIG‐seq first‐PCR primers comprising eight pairs of universal ISSR multiplex primers. Each reaction was carried out in a 7 μL volume containing 1.0 μL of template DNA, 3.5 μL of 2× multiplex PCR buffer, 0.035 μL of enzyme mix, 0.14 μL of each primer (10 μM), and 0.225 μL of nuclease‐free water. The PCR was performed in a 96‐well Thermal Cycler with the PCR amplification conditions of 94°C for 1 min and followed by 25 cycles of denaturation at 94°C for 30 s, annealing at 38°C for 1 min, extension at 72°C for 1 min, and a final extension at 72°C for 10 min.

Amplification success was verified using the Microchip Electrophoresis System (MultiNA, Shimadzu), with the DNA‐2500 Reagent Kit (Shimadzu). The first PCR products were purified prior to the second PCR.

The second PCR was performed with PrimeSTAR GXL DNA polymerase (Takara, RR050) reagent. Each reaction was carried out in a 12 μL volume containing 2.5.0 μL of diluted first PCR product, 2.4 μL of 5× PrimeSTAR GXL buffer, 0.96 μL of dNTP mixture, 1.2 μL of each primer (2 μM), 0.24 μL PrimerSTAR GXL polymerase, and 3.5 μL of nuclease‐free water. The second PCR was performed in a 96‐well Thermal Cycler using the following amplification profile: 98°C for 10 s for denaturation, 54°C for 15 s for annealing, and 68°C for 1 min for extension, repeated in 12 cycles.

The second PCR products from each sample were pooled in equal amounts and purified again, with a size‐selection step using AMPure XP magnetic beads (Beckman Coulter) to remove fragments shorter than 250 bp, ensuring the library consists of appropriately sized DNA fragments.

The pooled library was subjected to high‐throughput sequencing on an Illumina NextSeq 1000 platform using NextSeq 1000/2000 P1 Reagents (300 cycles). This enables the generation of substantial amounts of sequence data across many genomic regions.

### 2.4 Data Analyzes

#### 2.4.1 SNP Calling and Filtering

Raw MIG‐seq reads were processed using the de novo pipeline in Stacks v2.2 (Catchen et al. 2011). Loci were assembled and cataloged using cstacks, allowing a maximum of two mismatches between catalog loci (*n* = 2). Individual samples were then matched to the catalog using sstacks. Conversion of loci data to BAM format was performed using tsv2bam, followed by SNP calling and genotype likelihood estimation with gstacks (Catchen et al. 2011, 2013). Population‐level filtering was performed using a retention threshold of 0.5, retaining loci present in at least 50% of individuals across populations. The filtered dataset included 245 individuals and 1118 loci.

#### 2.4.2 Clone Detection and Diversity

Clonality can be biased in population genetics since it counts the same genotype multiple times (Arriesgado et al. 2015; Nakajima et al. 2017; Wainwright et al. 2018). Therefore, clone detection and correction were performed prior to downstream population genetic analyzes. The study used GenoDive v3.06 (Meirmans 2020) following Meirmans and Van Tienderen (2004). Individuals sharing identical allelic profiles across all single‐nucleotide polymorphism (SNP) loci were assigned to the same multilocus genotype (MLG), whereas individuals with differing alleles were considered distinct genets. Clone assignment was conducted separately for each population using a threshold of five allele differences. This threshold accounts for potential genotyping errors and somatic mutations while preventing overestimation of clonal diversity. Across all populations, the analysis included 245 samples and 1118 loci, with clone assignment identifying 156 distinct MLGs (see Table 2 for sample sizes per population).

**TABLE 2:** Clonal structure and richness of *H. uninervis* populations inferred using GenoDive v3.06 with a retention threshold of 5.

Population	Population size (*n*)/ramets	Distinct multilocus genotypes/genets (G)	Number of clonal repeats	Clonal richness (R)
Surigao	25	12	13	0.458
Nasipit	21	21	0	1.000
Mambajao	25	6	19	0.208
Laguindingan	24	2	22	0.043
Kauswagan	22	16	6	0.714
Plaridel	24	24	0	1.000
Maasin	25	24	1	0.958
Jagna	27	23	4	0.846
Maria	27	26	1	0.962
Sagay	25	2	23	0.042

*Note:* Clone assignment was based on identical allelic profiles across all SNP loci.

Clonal richness was calculated as *R* = (*G* − 1)/(*N* − 1), where *G* is the number of distinct MLGs, and *N* is the number of sampled individuals (Dorken and Eckert 2001). GenoDive computed standard clonal diversity indices, including the number of distinct genotypes, effective number of genotypes, Simpson's clonal diversity, genotypic evenness, and Shannon's diversity, corrected and uncorrected. Population differences in clonal diversity were tested using 1000 bootstrap replicates, and the variability of diversity estimates was assessed by jackknifing across loci to obtain standard deviations. Statistical significance was evaluated at *a* = 0.05.

Clone correction substantially reduced the number of individuals available for some populations, which may influence the resolution and stability of downstream analyzes such as Principal Component Analysis (PCA) and ADMIXTURE. However, clone correction was necessary to reduce biases associated with clonal pseudo‐replication and overrepresentation of identical multilocus genotypes in population genetic analyzes. All subsequent genetic analyzes were performed using this clone‐corrected dataset, retaining one sample per clone.

#### 2.4.3 Population Genetic Diversity

The geographical population genetic diversity was assessed using a clone‐corrected dataset derived from GenoDive clone assignment. The dataset was then reprocessed using the same Stacks v2.2 pipeline to generate a clone‐corrected SNP dataset. Diversity indices, including nucleotide diversity (*π*), heterozygosity (*H*_0_), and inbreeding coefficient (*F*_IS_), were extracted and summarized per population using a custom *R* bootstrap resampling pipeline to compute mean estimates and 95% confidence intervals (Coulon 2010). This resampling approach is particularly appropriate for datasets with modest sample sizes and has been shown to provide reliable estimates of genetic variability (Nazareno et al. 2017). Additionally, nucleotide diversity (*π*) and Tajima's *D* were computed from the variant call format (VCF) files using VCFtools v0.1.14 to investigate patterns of neutral evolution and potential selection across populations (Danecek et al. 2011).

#### 2.4.4 Population Genetic Structure

The genetic structure was analyzed using a clone‐corrected SNP dataset through Principal Component Analysis (PCA) with the adegenet v2.1.1 package in R v4.3.1 through RStudio v2023.9.0.463, to detect genetic patterns (Jombart 2008; Jombart and Ahmed 2011; Posit Team 2023). Analyzes were performed on a clone‐corrected SNP dataset comprising 156 MLGs representing 10 populations. PCA was conducted using the dudi.pca() function from the ade4 package, retaining the first 50 principal components (Bougeard and Dray 2018). Genetic structure among populations was visualized using scatterplots of the first two principal components (PC1 and PC2), which explain the largest proportions of genomic variance, with population clustering illustrated using 95% confidence ellipses generated in the ggplot2 package (Francis 2017) in R v4.3.1. In parallel, the population structure was inferred using the ADMIXTURE maximum‐likelihood algorithm (Alexander et al. 2009). The analyzes were performed for *K* = 1–10 with 20 independent replicates of each *K* under a 10‐fold cross‐validation scheme. Each replicate was initialized using distinct random seeds to test the stability of ancestry assignments. The optimal *K* was selected based on the lowest cross‐validation error in combination with consistency among replicate runs and biological interpretability of the inferred structure.

#### 2.4.5 Genetic Differentiation Between Populations

The genetic differentiation between populations was analyzed using the R package mmod v1.3.3 (Winter 2012). Analyzes were conducted on the clone‐corrected dataset, retaining one representative per multilocus genotype per population to avoid bias from clonal replicates. The study calculated three complementary estimators, including Nei's *G*_ST_ as a frequency‐based analogue of Wright's *F*_ST_ (Nei 1973), Hedrick's standardized *G*′_ST_ used to compare across loci with different levels of heterozygosity (Hedrick 2005), and Jost's *D* used for the diversity‐based measure of allelic differentiation (Jost 2008). Pairwise estimates were computed between all populations. Confidence intervals of 95% were generated by non‐parametric bootstrapping over loci with 1000 replicates (Winter 2012). For each replicate, loci were resampled with replacement, and all pairwise statistics were recalculated with 2.5% and 97.5% quantiles of the bootstrap distributions were used to derive confidence intervals for each population pair. Differentiation matrices were visualized as heatmaps for comparison of spatial clustering patterns and identification of highly differentiated populations.

#### 2.4.6 Isolation by Distance

The isolation by distance was analyzed by using the Mantel test in R v4.3.1, correlating pairwise genetic distance matrices derived from the clone‐corrected dataset, including Nei's *G*_ST_, Hedrick's *G*′_ST_, and Jost's *D*, with geographic distances among populations. Sample‐level geographic coordinates were averaged to obtain representative latitude and longitude values for each population. Geographic distances were calculated as Euclidean (straight‐line) distances between populations using a planar distance approximation (Hijmans 2024). Mantel tests were conducted using Pearson's correlation coefficient with 999 to assess the significance of the relationship between genetic and geographical distances (Jombart 2008; Jombart and Ahmed 2011).

#### 2.4.7 Recent Migration Inference

Recent migration among populations was assessed using BayesAss v3.0.4 (Wilson and Rannala 2003) based on a clone‐corrected dataset, which estimates contemporary gene flow rates occurring within the last 1 to 5 generations without assuming drift‐migration equilibrium (Faubet et al. 2007; Mussmann et al. 2019). The SNP dataset was randomly reduced to 500 loci using a custom R script, retaining 156 cloned‐corrected individuals across 10 populations. This subsampling approach minimizes potential linkage among loci and reduces the computational burden associated with large SNP datasets, while maintaining sufficient genetic signal for reliable estimation of recent migration rates. Three independent Markov Chain Monte Carlo (MCMC) runs were performed using 21,000,000 iterations. Convergence across runs was assessed by visual inspection of likelihood traces. Migration rates and flow were visualized using annotated heatmaps and geographic flow diagrams in RStudio v2023.9.0.463, facilitating comparison of relative migration intensity and direction of contemporary gene flow among populations. For visualization of the migration network, only migration rates within the range of ≥ 0.025 and ≤ 0.040 were retained. This threshold was applied to highlight moderate and potentially biologically meaningful migration rates while minimizing the inclusion of very low‐probability estimates that may reflect background noise or estimation uncertainty. This filtering does not affect the underlying migration rates, but improves interpretability by emphasizing dominant connectivity pathways among populations.

## 3 Results

### 3.1 Clone Detection and Diversity

Out of 245 extracted samples, there are 1118 loci detected from GenoDive analysis using a retention threshold of 5 across 10 populations. Clonal structure varied among populations (Table 2), revealing a total of 156 distinct MLGs (genets). Highly clonal populations such as Sagay (*G* = 2 of *N* = 25), Laguindingan (*G* = 2 of *N* = 24), and Mambajao (*G* = 6 of *N* = 25) were dominated by a small number of genets, resulting in very low clonal richness (*R* = 0.04, 0.21). In contrast, Nasipit and Plaridel exhibited no clonal repetition (*G* = *N*), indicating predominantly sexual recruitment. Intermediate levels of clonality were observed in Kauswagan (*R* = 0.71) and Surigao (*R* = 0.46). Clonal diversity indices further reflected these patterns (Table 3). The high clonal diversity was observed in Plaridel, Nasipit, Maasin, Jagna, and Maria, where nearly all sampled individuals possessed distinct MLGs. Moderate diversity was observed in Surigao and Kauswagan, having nearly half of the sampled individuals being distinct MLGs, while low diversity was characterized in Laguindingan, Sagay, and Mambajao, showing only a few MLGs dominating the population. A randomization test using the corrected Nei's diversity index revealed significant deviations from random mating in most populations (*p* < 0.05). Observed clonal diversity was significantly lower than expected under random mating in most populations at *p* = 0.001, indicating widespread clonal structure. No significant differences were detected in Plaridel, Nasipit, and Sagay, where *p* = 1.000, which conformed to expectations of random mating (Table 4).

**TABLE 3:** The clonal diversity and indices of *H. uninervis* across all populations were generated by GenoDive v3.06, with a retention threshold of 5.

Population	Effective number of genotypes	Clonal diversity index (Simpson's index)	Genotypic evenness	Evenness index	Shannon index (corrected for sample size)	Shannon index (uncorrected)
Surigao	5.631	0.857	0.822	0.469	1.127	0.916
Nasipit	21.000	1.000	0.952	1.000	nan	1.322
Mambajao	1.543	0.367	0.352	0.257	0.554	0.357
Laguindingan	1.180	0.159	0.153	0.590	0.137	0.125
Kauswagan	8.963	0.931	0.888	0.560	1.547	1.103
Plaridel	24.000	1.000	0.958	1.000	nan	1.380
Maasin	23.148	0.997	0.957	0.965	2.479	1.374
Jagna	19.703	0.986	0.949	0.857	1.933	1.334
Maria	25.138	0.997	0.960	0.967	2.547	1.409
Sagay	1.083	0.080	0.077	0.542	0.120	0.073

*Note:* Shannon diversity (corrected for sample size) was undefined (nan) for Nasipit and Plaridel, as all individuals possessed distinct MLGs, resulting in maximal genotypic diversity. Values are color‐scaled with dark colors representing the highest values and light colors representing the lowest values.

**TABLE 4:** Comparison of observed and expected diversity under random mating for each population based on the corrected Nei's diversity index, obtained using GenoDive v3.06.

Population	Observed corrected Nei's diversity index (div_obs)	Expected diversity under random mating (div_exp)	Significance of the difference (*p*)
Surigao	0.857	1.000	0.001*
Nasipit	1.000	1.000	1.000
Mambajao	0.367	0.988	0.001*
Laguindingan	0.159	0.805	0.001*
Kauswagan	0.931	1.000	0.001*
Plaridel	1.000	1.000	1.000
Maasin	0.997	1.000	0.001*
Jagna	0.986	1.000	0.001*
Maria	0.997	1.000	0.001*
Sagay	0.080	0.003	1.000

*Note:* Significant *p*‐values indicate deviation from random mating.

* *p* < 0.05 statistically significant.

### 3.2 Population Genetic Diversity

**FIGURE 2:**
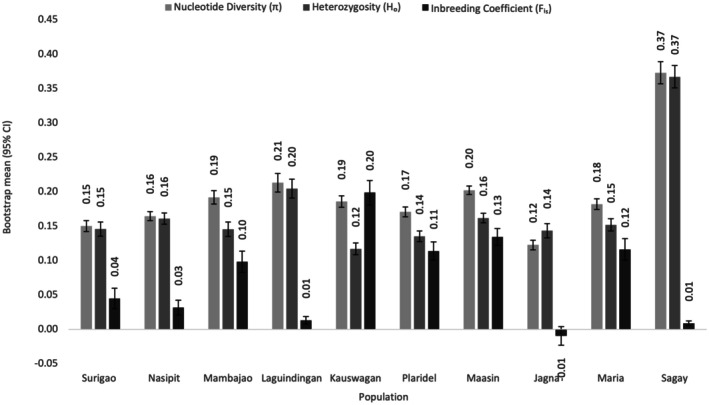
Mean nucleotide diversity (*π*), observed heterozygosity (*H*ₒ), and inbreeding coefficient (*F*_IS_) were estimated from a clone‐corrected SNP dataset generated using Stacks v2.2. Error bars represent 95% confidence interval obtained from bootstrap resampling across loci.

**FIGURE 3:**
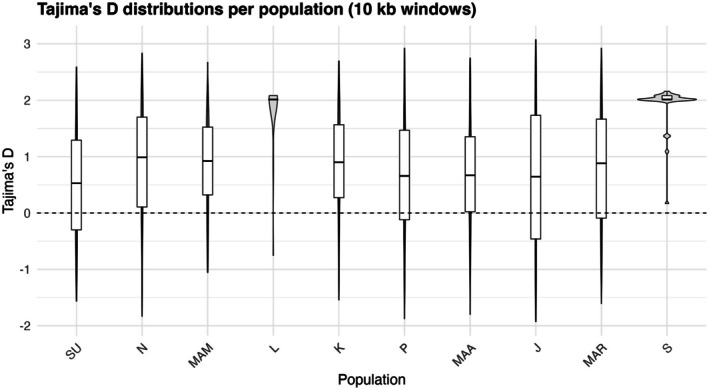
Distribution of Tajima's *D* values calculated from 10 kb non‐overlapping genomic windows for each population using VCF tools v0.1.14. Boxplots summarize population‐level variation in deviations from neutral expectations. SU = Surigao, *N* = Nasipit, MAM = Mambajao, L = Laguindingan, K = Kauswagan, *P* = Plaridel, MAA = Maasin, J = Jagna, MAR = Maria, and S = Sagay.

**FIGURE 4:**
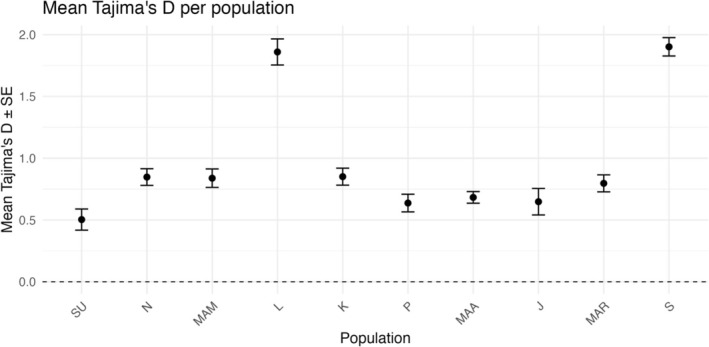
Mean of Tajima's *D* per population with standard error per population derived from 10 kb windows, showing the differences in allele frequency spectra among *H. uninervis* populations. SU = Surigao, *N* = Nasipit, MAM = Mambajao, L = Laguindingan, K = Kauswagan, *P* = Plaridel, MAA = Maasin, J = Jagna, MAR = Maria, and S = Sagay.

Genetic diversity analysis of clone‐corrected datasets showed nucleotide diversity (*π*) ranging from 0.12 in Jagna to 0.37 in Sagay. Observed heterozygosity (*H*_0_) varied from 0.12 in Kauswagan to 0.37 in Sagay, while the inbreeding coefficient (*F*_IS_) ranged from −0.01 in Jagna to 0.20 in Kauswagan. The Sagay population, as an outgroup, exhibited the highest genetic variability, whereas Jagna had the lowest (Figure 2). Tajima's *D* values calculated from 10 kb non‐overlapping windows across all populations ranged from approximately −2 to +3 (Figure 3). The mean Tajima's *D* per population ranged from 0.45 ± 0.08 in Surigao to 2.01 ± 0.10 in Sagay, with most populations exhibiting positive Tajima's *D* values. The highest mean values were observed in the Laguindingan and Sagay populations, while the lowest were detected in Surigao (Figure 4).

### 3.3 Population Genetic Structure

**FIGURE 5:**
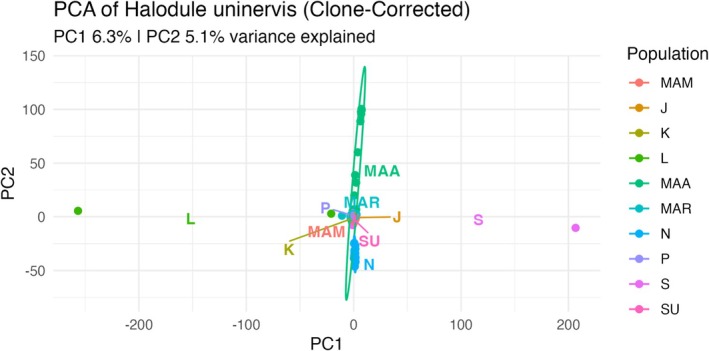
The first two principal components (PC1 = 6.3%, PC2 = 5.1%) of *H. uninervis* populations. Ellipses represent 95% confidence intervals for populations computed where *n* ≥ 3; Laguindingan and Sagay are shown as individual points due to small sample sizes. SU = Surigao, *N* = Nasipit, MAM = Mambajao, L = Laguindingan, K = Kauswagan, *P* = Plaridel, MAA = Maasin, J = Jagna, MAR = Maria, and S = Sagay.

**FIGURE 6:**
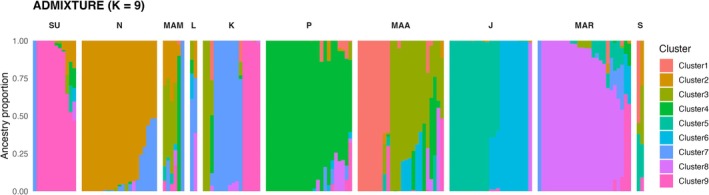
ADMIXTURE ancestry profiles of *H. uninervis* across 10 populations (K = 9), the model with lowest cross‐validation error (0.357). Interpretation of ancestry components should be made cautiously due to low differences among higher K values and reduced clone‐corrected sample sizes in some populations. SU = Surigao, *N* = Nasipit, MAM = Mambajao, L = Laguindingan, K = Kauswagan, *P* = Plaridel, MAA = Maasin, J = Jagna, MAR = Maria, and S = Sagay.

Principal Component Analysis (PCA) based on 6952 SNPs from the clone‐corrected datasets revealed the genetic relationships across the 10 populations. The first two principal components (PC1 = 6.3%, PC2 = 5.1%) indicate weak genetic structuring among populations. Populations from Bohol Sea formed partially overlapping clusters, suggesting limited differentiation and potential gene flow across sites (Figure 5). The Sagay population from the Visayan Sea showed a relatively tight cluster but remained near the Bohol Sea groups in ordination space. Because some populations had reduced clone‐corrected sample sizes, the observed clustering patterns should be interpreted cautiously. ADMIXTURE analysis was evaluated across multiple *K* values using cross‐validation error. The lowest CV error was observed at *K* = 9 (CV = 0.357). However, the difference among higher *K* values was relatively small, and therefore, *K* = 9 was interpreted cautiously as the most supported model. At *K* = 9, populations showed varying ancestry profiles, with some populations exhibiting partially shared ancestry components and others showing more homogeneous patterns (Figure 6). Among populations, Maasin, Mambajao, Laguindingan, Kauswagan, and Sagay showed relatively admixed ancestry profiles, whereas Surigao, Nasipit, Plaridel, Maria, and Jagna showed homogeneous ancestry patterns.

### 3.4 Genetic Differentiation Between Populations

**FIGURE 7:**
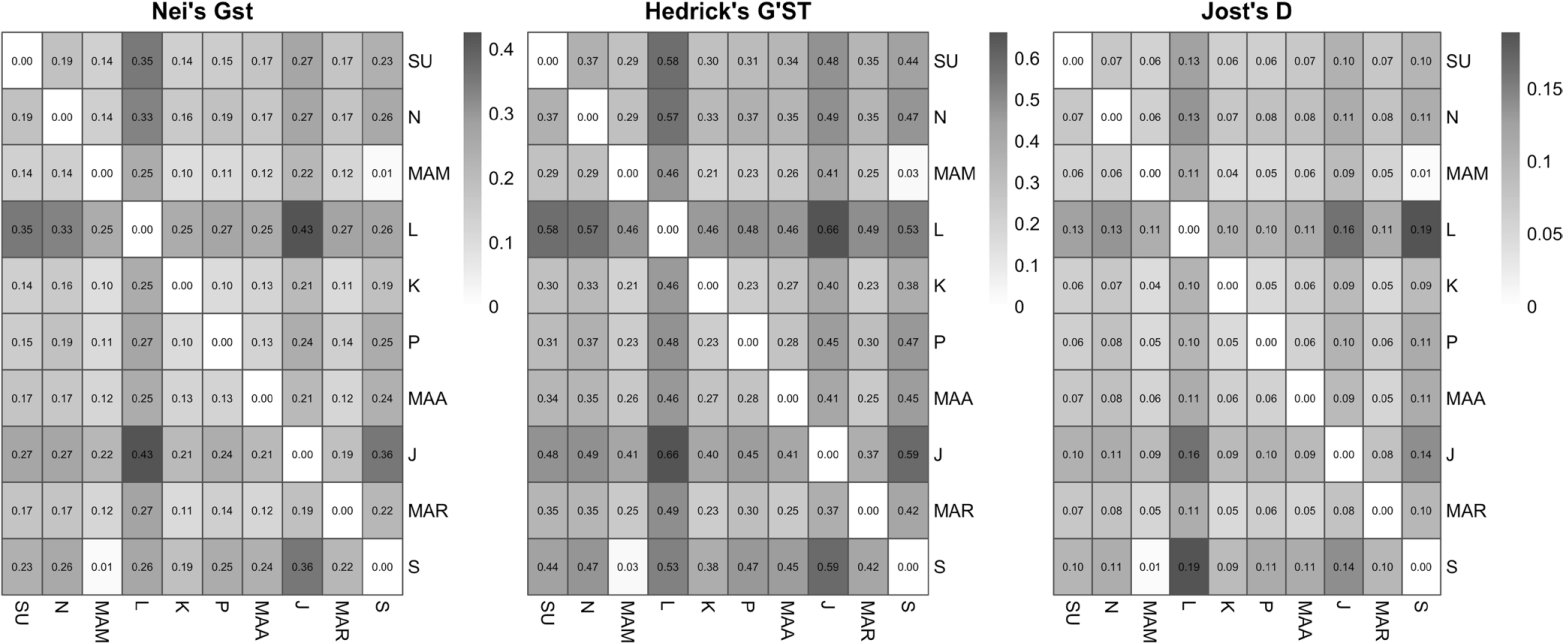
The three complementary metrics of genetic differentiation values of *H. uninervis* across all populations were calculated from the clone‐corrected SNP dataset, whereas darker shades indicate higher differentiation. SU = Surigao, *N* = Nasipit, MAM = Mambajao, L = Laguindingan, K = Kauswagan, *P* = Plaridel, MAA = Maasin, J = Jagna, MAR = Maria, and S = Sagay.

Genetic differentiation analyzes using Nei's *G*_ST_, Hedrick's *G*′_ST_, and Jost's *D* consistently revealed low to moderate genetic differentiation structure among the populations. All metric results showed the highest values for Laguindingan to Jagna, Jagna to Sagay, Surigao to Laguindingan, and Nasipit to Laguindingan. In contrast, the lowest differentiation values were detected in Sagay to Mambajao at 0.01 to 0.03 (Figure 7).

### 3.5 Isolation by Distance

**FIGURE 8:**
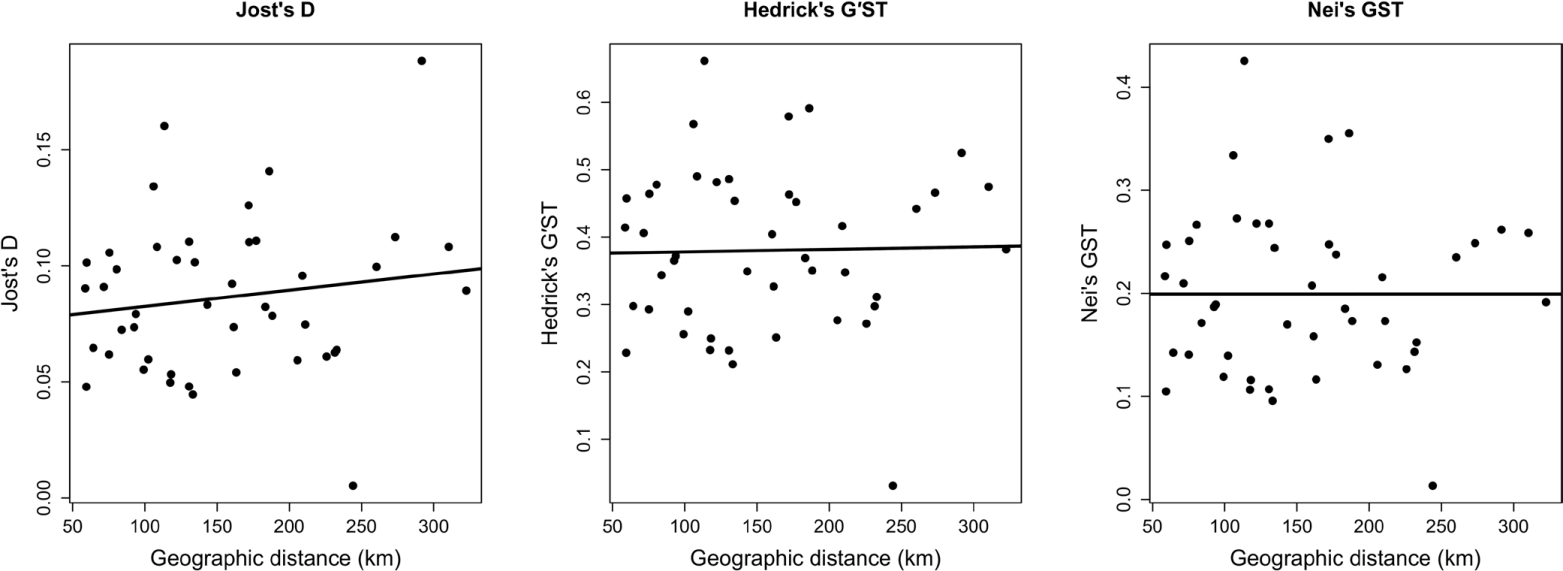
The relationship of pairwise geographic distance (km) and genetic differentiation metrics (Jost's *D*, Hedrick's *G*′_ST_, and Nei's *G*_ST_). Mantel tests based on 999 permutations revealed no significant correlations, indicating the absence of an IBD pattern among populations.

The pattern detected using Mantel tests with 999 permutations revealed no significant association between geographic distance and genetic differentiation among populations. Nei's *G*_ST_ and Hedrick's *G*′_ST_ exhibited no significant correlations at *r* = 0.000005, *p* = 0.466, and *r* = 0.0225, *p* = 0.427, respectively. Thus, Jost's *D* exhibited a weak positive correlation, but this relationship was not statistically significant at *p* = 0.269 (Figure 8, Table 5). These results indicate that genetic differentiation among populations was not strongly associated with the geographic distance across the study area, potentially reflecting the influence of oceanographic connectivity and dispersal process.

**TABLE 5:** Mantel test results of *H. uninervis* across populations assessing IBD using Jost's *D*, Hedrick's *G*′_ST_, and Nei's *G*_ST_ based on 999 permutations.

Genetic distance metric	Mantel *r*	*p*
Jost's *D*	0.1509	0.269
Hedrick's *G*′_ST_	0.0225	0.427
Nei's *G*_ST_	0.000005	0.466

*Note:* *p* < 0.05 statistically significant. No significant correlation detected.

### 3.6 Recent Migration Inference

**FIGURE 9:**
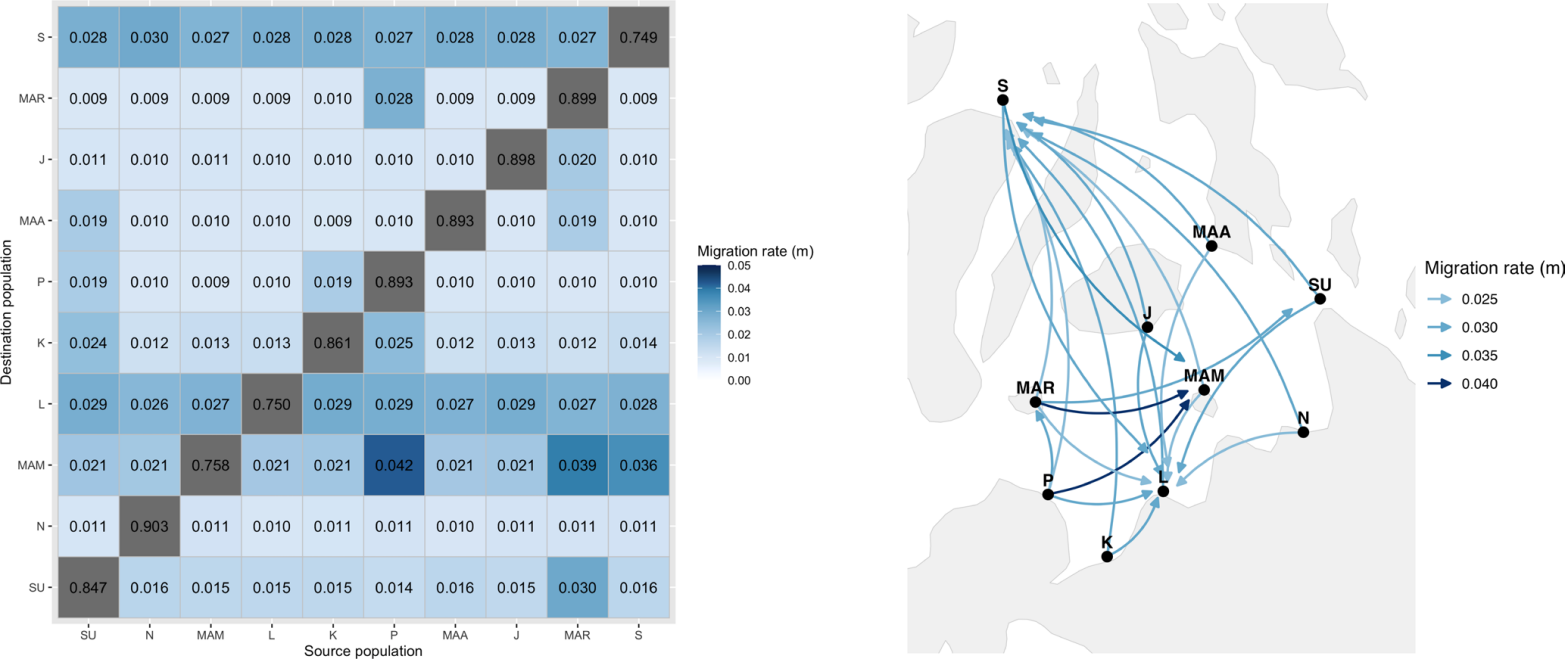
Heatmap (left) and migration network (right) of recent gene flow inferred by BayesAss. The heatmap illustrates recent migration rates, where darker blue shading indicates higher migration probabilities and gray shade represent self‐recruitment, while the migration network with only migration rates ≥ 0.025 and ≤ 0.040 is shown to highlight relatively strong network migration pathways. SU = Surigao, *N* = Nasipit, MAM = Mambajao, L = Laguindingan, K = Kauswagan, *P* = Plaridel, MAA = Maasin, J = Jagna, MAR = Maria, and S = Sagay.

The recent migration inference, which estimates contemporary gene flow occurring approximately one to five generations, revealed a generally high self‐recruitment, particularly in Nasipit, with a rate of 0.903, followed by Maria, Jagna, Maasin, Plaridel, Kauswagan, and Surigao, which ranged from 0.847 to 0.899. Moderately high self‐recruitment was observed at Sagay, Laguindingan, and Mambajao, ranging from 0.749 to 0.758. The highest estimated migration rate was observed from Plaridel to Mambajao at 0.042, followed by Maria to Mambajao, Sagay to Mambajao, and Maria to Surigao at 0.039, 0.036, and 0.030, respectively. Moderation migration estimates were also observed toward Laguindingan and Sagay. In contrast, Nasipit, Kauswagan, Plaridel, Maasin, Jagna, and Maria exhibited lower estimated incoming and outgoing migration rates (Figure 9). However, these migration estimates should be interpreted cautiously because reduced clone‐corrected sample sizes in some populations may affect the precision and stability of recent migration inference.

## 4 Discussion

### 4.1 Clone Detection and Diversity

The reproductive strategies of *H. uninervis* populations exhibit spatial variation, ranging from predominantly sexual reproduction to strongly clonal propagation. Populations such as Plaridel, Nasipit, Maasin, Jagna, and Maria showed high clonal richness and clonal diversity values, suggesting frequent sexual recruitment and the presence of genetically distinct individuals (Arriesgado et al. 2023a). The absence of significant deviation from random mating in Plaridel and Nasipit further supports the interpretation that sexual recruitment contributes substantially to genetic diversity in these populations (Arriesgado et al. 2023b; Malanguis et al. 2024). In contrast, low clonal richness and diversity values, such as population in Laguindingan, Sagay, and Mambajao, reflect strong clonal propagation and dominance of a few genets (Litsi‐Mizan et al. 2023). Moderate clonal richness in Surigao and Kauswagan suggests mixed reproductive strategies involving both clonal and sexual recruitment.

Although Sagay showed no significant difference between observed and expected diversity under random mating, both observed and expected diversity values remained relatively low. This emphasizes the importance of interpreting random mating tests with absolute diversity metrics, as conformity to expectations does not necessarily imply high genetic diversity (Arriesgado et al. 2023a, 2023b). However, interpretations involving populations with small clone‐corrected sample sizes should be made cautiously, as reduced sample representation may influence diversity estimates and detection of clonal structure.

Differences in clonal structure among populations may reflect variation in local environmental conditions, disturbances, and connectivity patterns. High clonal richness populations, such as Plaridel, Nasipit, Maasin, Jagna, and Maria, are located in a hydrodynamically connected coastal environment with minimal disturbance that may support recurrent recruitment and dispersal processes (Evans et al. 2021). In contrast, low clonal richness populations, such as Laguindingan, Sagay, and Mambajao, may experience localized habitat disturbances, which limit seedling establishment and may favor the clonal expansion of a limited number of genets (McMahon et al. 2017; Dierick et al. 2021). Moderate clonal richness populations, such as Surigao and Kauswagan, may reflect intermediate reproductive dynamics involving both clonal and periodic sexual recruitment (McMahon et al. 2017).

Similar patterns have been commonly reported in seagrass ecosystems, where reproductive structure is influenced by the interaction of local environmental conditions, disturbance regimes, and hydrodynamic processes (e.g., Paulo et al. 2019; Xu et al. 2019; Jiang et al. 2020; Johnson et al. 2020; Arriesgado et al. 2023a). Intermediate disturbances may facilitate coexistence between clonal propagation and sexual recruitment, whereas stronger or more persistent disturbances can reduce seedling establishment and favor persistence of established clones (McMahon et al. 2017). In contrast, frequent and intense physical disturbance may act as a recruitment bottleneck by reducing seedling survival and dominating the long‐term survival of established clones (Dierick et al. 2021). Oceanographic circulation and seasonal current variability may also contribute to dispersal and connectivity among populations, although these processes were not directly evaluated in the present study. The observed clonal structure reflects varying reproductive strategies and population dynamics across sites, while emphasizing the importance of clone correction in minimizing pseudo‐replication in population genetic analyzes.

### 4.2 Population Genetic Diversity

The genetic diversity of *H. uninervis* populations generally exhibits low to moderate variability, as reflected by relatively low nucleotide diversity (*π*) and observed heterozygosity (*H*ₒ) at most sites. These patterns were consistent with previous studies on seagrasses, where extensive clonal propagation and uneven sexual recruitment contribute to localized genetic structure (Evans et al. 2021; Hernawan et al. 2023). Among the populations, Sagay displayed comparatively higher genetic variability, whereas Jagna showed the lowest nucleotide diversity, suggesting reduced genetic variation relative to the other populations (Nguyen et al. 2014, 2021; Alias et al. 2024). However, interpretations involving populations with reduced clone‐corrected sample sizes should be made cautiously, as diversity estimates and demographic statistics may be sensitive to sample representation.

Despite its low nucleotide diversity (*π*), Jagna showed a slightly negative inbreeding coefficient (*F*_IS_). However, this pattern does not imply enhanced sexual reproduction or the absence of inbreeding (Xu et al. 2019; Hernawan et al. 2023). Thus, it reflects heterozygote excess at a small number of polymorphic loci, a statistical outcome that can arise when few remaining genets are heterozygous and overall allelic variation is low. Similar patterns have been reported in *Halophila ovalis* and may arise when relatively few heterozygous genets persist within populations with low allelic diversity (Xu et al. 2019). In contrast, positive *F*_IS_ values observed in most populations suggest heterozygote deficiency, which may be associated with clonal growth, localized dispersal, and population subdivision (Evans et al. 2021, Hernawan et al. 2023).

Most populations exhibited positive mean Tajima's *D* values, indicating an excess of intermediate‐frequency alleles relative to neutral expectations. Such a pattern may reflect balancing selection, population subdivision, and population contraction, rather than strict neutral evolution (Schmidt and Pool 2002). Populations with the highest mean Tajima's values, such as Laguindingan and Sagay, suggest more complex demographic or reproductive histories relative to the other populations (Hernawan et al. 2023). In Sagay, the combination of relatively high genetic diversity and positive Tajima's *D* may indicate the persistence of multiple genetic variants within the population, potentially influenced by historical demographic processes and limited contemporary connectivity (Hernawan et al. 2023; Litsi‐Mizan et al. 2023). However, these interpretations remains tentative because the underlying demographic history was not directly tested in this study.

In contrast, Laguindingan exhibited lower genetic diversity together with positive Tajima's *D* values, which may reflect reduced allelic turnover under strong clonal dominance of historical demographic changes (Evans et al. 2021, Hernawan et al. 2023). Surigao exhibited the lowest mean Tajima's *D*, closer to neutral expectations, which may reflect comparatively recent population expansion or the recovering stage after disturbance (Evans et al. 2021). The contrasting Tajima's *D* patterns among populations suggest heterogeneous demographic and reproductive dynamics across sites, potentially influenced by local environmental conditions, historical connectivity, and reproductive strategies (Pazzaglia et al. 2021; Hernawan et al. 2023).

### 4.3 Population Genetic Structure

The genetic structure of *H. uninervis* across the regions revealed weak genetic structuring, consistent with high connectivity and ongoing gene flow. However, the relatively low proportion of variance explained by the first two principal components indicates that the observed clustering patterns should be interpreted cautiously. Such low PCA values are commonly reported in marine foundation species with large effective population sizes and extensive dispersal, where genetic variation is distributed across many loci rather than concentrated along a few major components (Hernawan et al. 2017; Hosokawa et al. 2025; Song et al. 2025). In addition, reduced clone‐corrected sample sizes in some populations may have limited the resolution of clustering analyzes and ancestry assignment patterns.

Most populations within Bohol Sea formed partially overlapping clusters in principal component ordination, suggesting limited differentiation and partially shared genetic backgrounds (Arriesgado et al. 2023a; Hernawan et al. 2023). Similar patterns have also been reported in tropical Indo‐Pacific seagrass populations, where genetic structure reflects a combination of historical connectivity and ongoing gene flow (e.g., Hernawan et al. 2017; Arriesgado et al. 2023a). Nevertheless, given the low explanatory power of PCA, these patterns should not be overinterpreted as definitive evidence of population connectivity.

The Sagay population formed a relatively tight cluster, indicating lower within‐population variation, but remained positioned close to Bohol Sea populations, suggesting some degree of genetic affinity (Hosokawa et al. 2025). This pattern may reflect partial geographic separation combined with historical or episodic connectivity (Arriesgado et al. 2023b; Hosokawa et al. 2025).

ADMIXTURE analysis showed varying ancestry compositions across populations at *K* = 9 (CV = 0.357), although differences among higher K values were relatively small. Thus, *K* = 9 should be interpreted cautiously as the most supported clustering model. Populations such as Maasin, Mambajao, Laguindingan, Kauswagan, and Sagay showed high admixture proportions, indicating the contribution of multiple ancestral sources, while Surigao, Nasipit, Plaridel, Maria, and Jagna exhibited more homogeneous ancestry profiles (Hernawan et al. 2023). The observed population structure likely reflects the combined influence of localized recruitment, partial connectivity, and shared evolutionary history across the region (Hernawan et al. 2023; Hosokawa et al. 2025).

### 4.4 Genetic Differentiation Between Populations

Genetic differentiation analyzes consistently indicated low to moderate levels of genetic differentiation among *H. uninervis* populations, which strengthens the inference that population structure is present but relatively weak (Arriesgado et al. 2023a). Similar patterns have been reported in the tropical Indo‐Pacific, where moderate differentiation occurs despite evidence of partial connectivity among sites (Hernawan et al. 2017; Arriesgado et al. 2023a). However, interpretations of differentiation patterns should be made cautiously because some populations had reduced clone‐corrected sample sizes, which may influence estimates of genetic structure.

The highest differentiation values were observed between Laguindingan and Jagna (~114 km), Jagna and Sagay (~186 km), Surigao and Laguindingan, and Nasipit and Laguindingan (~105 km). These patterns suggest that Laguindingan may be comparatively differentiated from several populations. Such differentiation could be associated with localized environmental conditions, habitat discontinuity, or reduced effective connectivity at local scales rather than complete genetic isolation (Jackson et al. 2020; Hosokawa et al. 2025).

In contrast, the relatively low differentiation values were detected between Sagay and Mambajao, despite these populations being separated by approximately 243 km. This pattern may reflect partial history connectivity, shared ancestry, or episodic dispersal processes across broader scales. Oceanographic circulation, monsoon‐driven currents, and the presence of intermediate habitats could potentially facilitate dispersal of propagules and vegetative fragments among populations, although these processes were not directly evaluated in the present study (Hernawan et al. 2023; Nakajima et al. 2023; Hosokawa et al. 2025).

### 4.5 Isolation by Distance

Mantel test analyzes revealed no significant association between geographic distance and genetic differentiation across all the metrics, including Nei's *G*_ST_, Hedrick's *G*′_ST_, and Jost's *D*, indicating the absence of a clear isolation‐by‐distance (IBD) pattern in *H. uninervis* population (Xuereb et al. 2018; Jahnke and Jonsson 2022; Hosokawa et al. 2025). These results suggest that geographic distance alone does not explain genetic differentiation within the study area (Hernawan et al. 2023). However, interpretations should be made cautiously because some populations have reduced clone‐corrected sample sizes, which may influence estimates of genetic structure and connectivity.

Several ecological and oceanographic factors may contribute to the weak IBD pattern observed in this study. The Bohol Sea is characterized by complex circulation dynamics, including the Bohol Jet and localized eddies such as Iligan Bay, which can facilitate multidirectional dispersal of propagules and vegetative fragments across spatially distant sites. These circulation features may enhance connectivity beyond simple geographic proximity. In addition, the presence of multiple coastal habitats and island systems across the region may promote stepping‐stone dispersal, allowing gradual gene flow among populations even over relatively large distances. Such processes can reduce the effect of geographic distance on genetic differentiation (Evans et al. 2021; Jahnke and Jonsson 2022; Hernawan et al. 2023; Legrand et al. 2024; Hosokawa et al. 2025).

The biological characteristics of *H. uninervis* further support potential long‐distance dispersal. As a species capable of both sexual and asexual reproduction, it can disperse via seeds, floating fragments, and detached shoots that remain viable during transport, thus increasing successful colonization (Evans et al. 2021). Similar patterns of weak or non‐significant IBD have been reported in other tropical Indo‐Pacific regions, where dispersal processes and local habitat configuration may reduce the influence of geographic distance on genetic differentiation (Hernawan et al. 2017, Arriesgado et al. 2023a).

Nevertheless, these interpretations remain tentative because the Mantel test used Euclidean (straight‐line) geographic distances, which do not account for actual dispersal pathways influenced by ocean currents and coastal configuration. Thus, the absence of significant IBD observed in this study may partly reflect limitations of the geographic distance model used. Future analyzes incorporating oceanographic distance models or biophysical dispersal simulations would help clarify the relative contribution of the oceanographic processes to connectivity patterns in *H. uninervis* populations.

### 4.6 Recent Migration and Connectivity

BayesAss analyzes revealed generally high self‐recruitment rates across most populations, indicating high local retention of individuals. Nasipit showed the highest self‐recruitment, whereas several other populations showed similarly elevated values. A comparable pattern was reported in corals and reef fishes, where local reproduction plays a major role in maintaining population genetic composition despite evidence of broader regional connectivity (López‐Márquez et al. 2019; Swearer et al. 2020; Afiq‐Rosli et al. 2021).

Despite high self‐recruitment, asymmetric migration estimates were detected among several population pairs. In particular, Mambajao showed comparatively higher incoming migration from Plaridel (~130 km), Maria (~117 km), and Sagay (~243 km), whereas Surigao also received moderate migration estimates from multiple populations. These patterns may suggest relatively greater connectivity with neighboring populations compared to other sites, although interpretations should remain cautious because migration estimates may be influenced by reduced clone‐corrected sample sizes and assumptions of the BayesAss model.

In contrast, populations such as Nasipit, Kauswagan, Plaridel, Maasin, Jagna, and Maria exhibited relatively low incoming and outgoing migration rates, which may reflect more localized recruitment dynamics (López‐Márquez et al. 2019; Swearer et al. 2020). The combination of high self‐recruitment and selective migration estimates is consistent with partially connected dynamics, where gene flow occurs through specific pathways influenced by local recruitment, habitat distribution, and stochastic dispersal processes (López‐Márquez et al. 2019; Swearer et al. 2020; Afiq‐Rosli et al. 2021; Legrand et al. 2024).

Oceanographic circulation in the Bohol Sea, particularly the Bohol Jet and localized eddy systems, can potentially facilitate episodic long‐distance dispersal of biological particles across the region (Bernardo 2011). However, these oceanographic processes were not directly evaluated in the present study, and therefore their contribution to the inferred migration patterns remains tentative. In addition, the migration network visualization was based on threshold‐filtered migration estimates (≥ 0.025 and ≤ 0.040) to emphasize moderation patterns and improve interpretability. Lower migration estimates not shown in the network may still contribute to overall connectivity patterns.

## 5 Conclusion

In conclusion, this paper provides a population genetic assessment of *Halodule uninervis* in the Bohol Sea and adjacent waters in the Philippines using clone‐corrected SNP datasets. The results revealed a spatial variation in reproductive strategies from predominantly sexual populations with stronger clonal propagation. Genetic diversity across all populations was generally low to moderate, while population structure analyzes indicated weak but detectable genetic differentiation among sites. Although some populations exhibited partially shared ancestry and migration estimates suggested selective connectivity among sites, the relatively low PCA variance and reduced clone‐corrected sample sizes in some populations indicate that these patterns should be interpreted cautiously.

No significant isolation‐by‐distance pattern was detected, suggesting that geographic distance alone may not fully explain the observed genetic structure. Oceanographic circulation, habitat configuration, and species dispersal characteristics can potentially contribute to connectivity patterns across the region, although these processes were not directly tested in this study. Migration analyzes further suggested generally high self‐recruitment together with variable levels of inferred connectivity among populations. Mambajao exhibited comparatively higher incoming migration estimates relative to several other populations. However, its role in regional connectivity remains tentative and requires further investigation.

These findings highlight the importance of considering local recruitment dynamics and regional connectivity in understanding the population structure of *H. uninervis*. These results provide baseline genetic information that may support future conservation, monitoring, and restoration planning for tropical seagrass ecosystems in the Philippines and other Indo‐Pacific regions. Future studies incorporating larger sample sizes, oceanographic modeling, and temporal sampling would help clarify the mechanisms of the connectivity patterns in the region.

## Author Contributions

**Angela Grace E. Singson:** conceptualization (lead), formal analysis (lead), investigation (lead), methodology (supporting), visualization (lead), writing – original draft (lead), writing – review and editing (equal). **Koji Takayama:** methodology (supporting), resources (lead), validation (supporting), writing – review and editing (equal). **Yoshihisa Suyama:** formal analysis (supporting), methodology (lead), resources (supporting), validation (lead), writing – review and editing (equal). **Naoko Ishikawa:** data curation (lead), formal analysis (supporting), methodology (supporting), resources (supporting), writing – review and editing (equal). **Shoki Murakami:** investigation (supporting), resources (supporting), writing – review and editing (equal). **Venus E. Leopardas:** conceptualization (supporting), writing – review and editing (equal). **Nonillon M. Aspe:** conceptualization (supporting), resources (supporting), writing – review and editing (equal). **Wilfredo H. Uy:** conceptualization (supporting), writing – review and editing (equal). **Lilibeth P. Coronel:** data curation (supporting), formal analysis (lead), resources (supporting), visualization (supporting), writing – review and editing (equal). **Dan M. Arriesgado:** conceptualization (supporting), writing – review and editing (equal). **Ruby C. Gonzales:** conceptualization (supporting), writing – review and editing (equal).

## Funding

This work was supported by Department of Science and Technology – Science, Technology, and Research for National Development (DOST–STRAND); Nagao Natural Environment Foundation (FY 2025–2028) under the leadership of Katsuyuki Eguchi and Kawatabi Field Science Center, Graduate School of Agricultural Science, Tohoku University.

## Conflicts of Interest

The authors declare no conflicts of interest.

## Data Availability

Raw sequencing reads generated in this study have been deposited in the NCBI Sequence Read Archive (SRA) under BioProject accession PRJNA1438458 [https://dataview.ncbi.nlm.nih.gov/object/PRJNA1438458?reviewer=p352mt27fg0qu7p6pp61hm1odb]. Codes and analytical workflows supporting this study are archived in Zenodo at [https://doi.org/10.5281/zenodo.20082471]. Source code is additionally maintained in GitHub for version control and development purposes: [https://github.com/angelagracesingson‐coder/Population‐genetics].
